# REAP-2: An interactive quantitative tool for robust and efficient dose-response curve estimation

**DOI:** 10.1017/cts.2023.642

**Published:** 2023-10-13

**Authors:** Xinying Fang, Xinyi Liu, Vernon M. Chinchilli, Michael Wang, Hong-Gang Wang, Nikolay V. Dokholyan, Chan Shen, J. Jack Lee, Shouhao Zhou

**Affiliations:** 1 Department of Public Health Sciences, Pennsylvania State University, Hershey, PA, USA; 2 Department of Lymphoma and Myeloma, University of Texas MD Anderson Cancer Center, Houston, TX, USA; 3 Department of Pharmacology, Pennsylvania State University, Hershey, PA, USA; 4 Department of Pediatrics, Pennsylvania State University, Hershey, PA, USA; 5 Department of Biochemistry and Molecular Biology, Pennsylvania State University, Hershey, PA, USA; 6 Department of Surgery, The Pennsylvania State University, Hershey, PA, USA; 7 Department of Biostatistics, University of Texas MD Anderson Cancer Center, Houston, TX, USA

**Keywords:** Shiny, robust beta regression, dose-response estimation, median-effect equation, sigmoid function, IC^50^, ED^50^, cancer biology, drug potency, quantitative tool, software

## Abstract

REAP-2 is an interactive dose-response curve estimation tool for Robust and Efficient Assessment of drug Potency. It provides user-friendly dose-response curve estimation for *in vitro* studies and conducts statistical testing for model comparisons with a redesigned user interface. We also make a major update of the underlying estimation method with penalized beta regression, which demonstrates great reliability and accuracy in dose estimation and uncertainty quantification. In this note, we describe the method and implementation of REAP-2 with a highlight on potency estimation and drug comparison.

## Introduction

Appropriate and reliable description of dose-response of individual compounds is vital in many active research fields including pharmacology, anesthesiology, toxicology, environmental science, and agrochemistry [1]. In contemporary drug development, it also demands accurate assessment of dose-response relationship [2]. Nevertheless, it poses unique challenges in the empirical assessment. Due to limited knowledge in the planning stage of the *in vitro* experiments, often a significant proportion of data are extreme observations, with values close to either 0% or 100% of the response [3]. Using the standard estimation approaches, we may experience either a significant efficiency loss if removing these extreme data points or a significant decrease in accuracy in the presence of the extreme observations, causing unstable estimation and erroneous uncertainty quantification of the dose-response relationship [3].

To overcome the analytical challenge, we developed the Robust and Efficient Assessment of drug Potency (REAP) to estimate the median-effect equation based on the beta regression framework, along with a freely accessible, web-based R Shiny app [3]. REAP takes on the beta law to account for non-normality and heteroscedasticity [4] and minimizes the average density power divergence with a tuning parameter [5]. Compared to the standard regression-based assessment, REAP proves to be more robust and powerful to extreme observations when estimating the median-effect equation [3].

**Figure 1.**
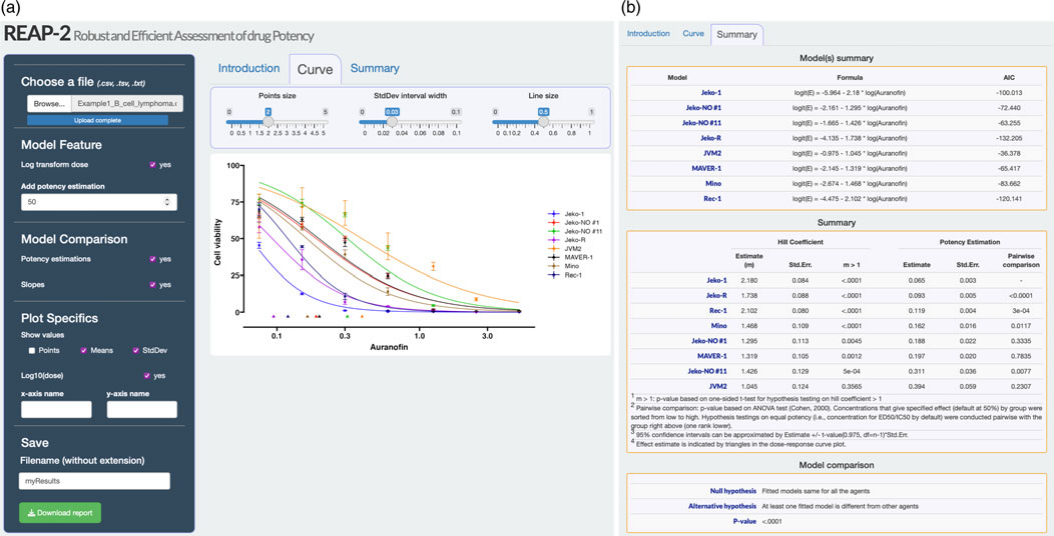
Overview of REAP-2 Shiny app. ***a***: Left panel and curve tab of REAP-2; ***b***: Summary tab of REAP-2.

Furthermore, to better assist the dose-response estimation, we conducted a comparative study [6] using a Monte Carlo simulation to review 14 different dose-response estimation tools including dose-response R packages and beta regression-based algorithms. We examined them in 30 different scenarios with various settings of extreme responses and concluded that in general, the penalized beta regression using the mgcv R package [7] had the best performance in terms of feasibility, accuracy, and coverage probabilities of the target estimands for dose-response. Therefore, as an improvement to REAP, we developed an updated version named REAP-2 and employed the penalized beta regression along with the *mgcv* package in REAP-2 for accurate and reliable estimation of the dose-response relationship (Fig. 1).

## Methods

### Median-Effect Equation for Dose-Response Curve

The median-effect equation, based on the mass-action law generalized from more than 300 mechanism-specific equations, is a unified theory in medicine to characterize the behavior of enzyme inhibitors [8,9]. It describe the *in vitro* dose-response relationship [10], and is formulated as:

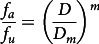




where *f*_*a*_ and *f*_*u*_ are the affected and unaffected cell fractions at the dose level *D*. *D*_*m*_ is the dose level, which generates the median effect. *m*, known as the Hill coefficient, describes the slope of the dose-response curve.

The median-effect equation can be simplified to:

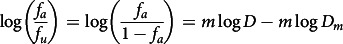




For statistical modeling, the equation can be rewritten as:

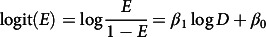




where *β*_0_ and *β*_1_ are the intercept and slope parameters that determine a sigmoid dose-response relationship with respect to the *E* = *f*_*a*_. The effect *E* is the expectation of the random variable *Y*. The observations of *Y* are used as the dependent variable to model the dose-response curve.

### Beta Regression

Beta regression is proposed to model bounded outcomes on the standard unit interval (0,1) (Ferrari and Cribari-Neto, 2004). The classical beta regression framework is built upon the mean (*μ*)-precision (*ϕ*) parameterization. Its density function is written as:

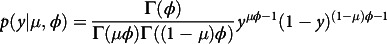




where *Γ*() denotes the gamma function, 0 < *μ* < 1 and *ϕ* > 0. The beta density is easy to interpret with mean and variance and it is flexible enough to take on a variety of shapes, accounting for non-normality and heteroscedasticity [4].

### Penalized Beta Regression with the mgcv Package

The estimation of the penalized beta regression is accomplished by the *mgcv* package [7]. It was developed to estimate the penalized generalized linear models by adding the L2 ridge penalty on the log-likelihood function with a tuning parameter. The parameter estimations are obtained by maximizing the penalized likelihood with the penalized iteratively re-weighted least squares program by *mgcv*.

## Implementation

### Overview of REAP-2

We redesigned the user interface of REAP [3] via Cascading Style Sheets and updated the estimation algorithm based on the comparative study results by implementing the penalized beta regression with the *mgcv* package [7] to estimate the dose-response.

REAP-2 Shiny app is freely available and accessible at https://xinying-fang.shinyapps.io/REAP/. The left panel of REAP-2 allows users to input data, specify the model and plot features, and download the analysis report (Fig. 1). The right panel contains three tabs: introduction, curve, and summary. The introduction tab provides an overview of REAP-2 as well as reference links to the previous version, REAP, and the manual of REAP-2. The curve tab (Fig. 1a) plots the dose-response curves and potency estimations based on the input data. The plot can be modified through Plot Specifics in the left panel and slider bars above the plot. The summary tab (Fig. 1b) provides table summaries on fitted median-effect equations, hill coefficients, potency estimations, and hypothesis testing for model comparison.

### Highlighted Features of REAP-2

#### Robust and efficient uncertainty quantification

REAP-2 applied penalized beta regression to dose-response estimation. The innovation not only ameliorates accuracy for the interested estimands, such as Hill coefficients and drug potency in both point and interval estimation, but also improves efficiency with narrower confidence intervals at the same nominal level. Most importantly, it largely enhances reliability of robust beta regression framework in dose-response estimation, despite various patterns of extreme data in real-life applications [6]. The results in previous comparative study demonstrate that the updated REAP-2 provides reliable estimations and possesses higher power in statistical testing.

#### Potency Estimation

REAP-2 enables estimations of self-specified compound potency (e.g., *IC*_*x*_ or *EC*_*x*_ values, corresponding to the concentrations that cause *x*% of the maximum inhibitory or maximum effect). REAP-2 enables the user-customization at any target effect of interest (*x*%, with *x* ranged in 0 and 100) and provides point estimation with standard deviation of the concentration to reach the target effect. The estimated concentrations are also represented as triangles in the dose-response curve plot (Fig. 1a). In the Summary tab (Second table of Fig. 1b), REAP-2 also enables pairwise comparison of potency estimations among different agents and conducts hypothesis testing on whether their potency estimations are the same.

## Availability and Future Direction

REAP-2 is accessible via a web interface at https://xinying-fang.shinyapps.io/REAP/. The source code and a user guide for REAP-2 are publicly available on GitHub at https://github.com/vivid225/REAP. It is built on an open-source platform within R Shiny environment and can be run under various operating systems such as Windows, Linux, and Mac systems. Additionally, it can be deployed in Docker and utilized as a web server.

REAP-2 is designed to be extensively used in everyday applications of data analysis and scientific reporting in drug screening for diverse non-computational scientists. The penalized beta regression employed in REAP-2 along with the Shiny app enable the robust and efficient dose-response estimation, which can be further integrated into methods to identify drug interaction effect.

In real-data application, there are a diverse selection of alternative nonlinear functional forms for dose-response modeling. Notably, a compilation of distinct nonlinear illustrations of dose-response relationships is comprehensively documented within the research conducted by Ritz *et al*. [11]. As a future extension of REAP-2, more nonlinear forms and model averaging could be employed to characterize the dose-response relationship.

## Data Availability

The REAP-2 Shiny app is available at https://xinying-fang.shinyapps.io/REAP-2/.
